# Enhanced Storage Stability of Different Polymer Modified Asphalt Binders through Nano-Montmorillonite Modification

**DOI:** 10.3390/nano10040641

**Published:** 2020-03-30

**Authors:** Zhibin Ren, Yongqiang Zhu, Qi Wu, Minye Zhu, Feng Guo, Huayang Yu, Jiangmiao Yu

**Affiliations:** 1 Research and Development Centre of Transport Industry of Technologies, Materials and Equipment of Highway Construction and Maintenance, Gansu Road and Bridge Group Co. Ltd., Lanzhou 730050, China; mszhibinren@mail.scut.edu.cn; 2 School of Civil Engineering and Transportation, South China University of Technology, Wushan Road, Tianhe District, Guangzhou 510000, China; 201730031412@mail.scut.edu.cn; 3 Key Laboratory for Special Area Highway Engineering, Chang’an University, Xi’an 710064, China; 4 Guangdong Guanyue Highway & Bridge Co., Ltd., Guangzhou 510000, China; ZHU15602229920@126.com; 5 Guangdong Province Communications Planning & Design Institute Co., Ltd., Xinghua Road, Tianhe District, Guangzhou 510000, China; wuqi614@126.com; 6 Department of Civil Engineering and Environment, University of South Carolina, Columbia, SC 29208, USA; fengg@email.sc.edu

**Keywords:** storage stability, rheological properties, polymer-modified asphalt, nano-montmorillonite

## Abstract

The storage stability concern, caused by phase separation for the density difference between polymers and asphalt fractions, has limited the widespread application of polymer modified asphalt (PMA). Therefore, this study aims to improve the storage concern of PMA by incorporating nano-montmorillonite. To this end, different nano-montmorillonites were incorporated to three PMAs modified with three typical asphalt modifiers, i.e., crumb rubber (CRM), styrene–butadiene-rubber (SBR) and styrene–butadiene-styrene (SBS). A series of laboratory tests were performed to evaluate the storage stability and rheological properties of PMA binders with nano-montmorillonite. As a consequence, the incorporation of nano-montmorillonite exhibited a remarkable effect on enhancing the storage stability of the CRM modified binder, but limited positive effects for the SBR and SBS modified binders. The layered nano-montmorillonite transformed to intercalated or exfoliated structures after interaction with asphalt fractions, providing superior storage stability. Among selected nano-montmorillonites, the pure montmorillonite with Hydroxyl organic ammonium performed the best on enhancing storage stability of PMA. This paper suggests that nano-montmorillonite is a promising modifier to alleviate the storage stability concern for asphalt with polymer modifiers.

## 1. Introduction

With the extremely increasing loading and aggravation of axis load in the pavement industry, damages such as rutting, cracking etc., are happening more frequently on highways and urban roads [1,2,3,4]. Asphalt modification technology has been considered a practical approach to resolve these concerns by enhancing the durability of asphalt pavements. Crumb rubber (CRM), styrene-butadiene-rubber (SBR) and styrene-butadiene-styrene (SBS) are the three most widely applied modifiers, which are regarded as effective adhesive and cohesive performance enhancers of asphalt [5,6,7,8,9]. However, the storage stability concern of modified asphalt has limited its widespread application. Engineers from asphalt plants have always worried about the separation of the modifier and asphalt during the storage and transportation process in elevated temperatures. 

According to Stoke’s law, the phase separation phenomenon can be governed by the following equation [10,11,12]:

(1) $$v_{t}=\frac{2a^{2}\Delta \rho g}{9\eta }$$

where *v_t_* is the settling velocity of dispersed particles, *α* is the radius of dispersed particles, Δ*ρ* is the density difference between two different phases, *g* is gravitational acceleration, and *η* is the dynamic viscosity of liquid medium.

Among different asphalt modifiers, CRM tends to sink in the liquid phase of modified asphalt during the storage process due to higher density compared to virgin asphalt, while SBR and SBS additives with lower density values tend to float in the upper part of liquid phase. The separation of modifier and raw asphalt results in a huge difference in composition and rheological properties between top and bottom portions of the polymer modified asphalt after storage. Previous studies indicate that this concern can be alleviated by adjusting the liquid asphalt density using bio-modification [13,14] or activating the crumb rubber [15]. However, the improvement effect is not very satisfactory. Therefore, one potential method is addressed in this study by incorporating nanoclay into the polymer-modified asphalt, which can reduce the phase separation phenomenon by decreasing the migration velocity of insoluble additive of polymers in the liquid phase [16,17].

Nanoclay is a type of natural mineral mainly including kaolinite clay (KC), vermiculite (VMT) and montmorillonite (MMT), which has a 2:1 layered structure with two silica tetrahedral sheets sandwiching an alumina octahedral sheet. Nowadays, nanomaterials have been popularly applied as modifiers for construction materials [12,18,19,20]. Especially, the importance of layered clay minerals, also known as nanoclays, in terms of asphalt modification is gradually increasing. Nanoclays’ remarkable improvement on the rheological properties of asphalt has been widely reported by previous studies. Vargas et al., [18] discovered that Organo-nanocomposite modified-asphalt can generate an intercalated structure using X-ray diffraction (XRD) and transmission electron microscopy (TEM). This indicates that the enhanced rheological properties may ascribe to the interaction behavior of the polymer chains in asphalt binder into the interlayer of clay. Yu et al., [21] enhanced the storage stability of asphalt rubber by incorporating three types of nanoclays and improved the rheological properties through modification. Leng et al., [22] found that clay/SBS modified bitumen composites have acceptable storage performance. The composites also showed better resistance to aging by reducing the oxidation of bitumen and the degradation of SBS. Galooyak et al., [23] proved the improvement of nanoclays on the storage stability of SBS modified asphalt and confirmed the conclusion through morphological analysis. Thus, nanoclay was expected by the asphalt industry to serve as the storage stability improver of PMAs. 

The objective of this study was to evaluate the feasibility of alleviating the storage stability concern of polymer-modified asphalt by incorporating nano-montmorillonite. To this end, three different types of nano-montmorillonites and three types of modifiers, i.e., CRM, SBR and SBS, were selected to prepare NPMAs. The rheological tests were performed to evaluate mechanical properties of NPMA binders when applied to pavement industry, among which the Superpave rutting factor test was chosen to control the content of these three modifiers. With the same PG82 grade, the content of modifier applied in each PMA binder was high enough to simulate the most unfavorable situation after storing in high temperature. In addition, the storage stability of modified binders was quantitatively analyzed through characterizing the differences in softening point, complex moduli and absorbance ratio of CRM/SBR/SBS in the infrared spectrum between the top and bottom portions of the sample after a lab-simulated storage process. Finally, an X-ray diffraction (XRD) test was conducted to investigate the layer gap distance variation of nano-montmorillonite to reveal the modification mechanism.

## 2. Materials and Methods

### 2.1. Materials and Sample Preparation

A Pen 60/70 virgin asphalt, with a penetration grade of 60/70, was used to prepare NPMA binders. In this study, all modified binders including polymer-modified asphalts, nano-montmorillonite-modified asphalts (NMA) and nano-montmorillonite-polymer modified asphalts were prepared by 10,000 rpm high shear incorporating modifiers with a certain dosage into virgin asphalt at 180 °C for 1 h. The selected dosages were 20 wt %, 7 wt % and 6 wt % by virgin asphalt for CRM (40-mesh), SBR and SBS, respectively, then a certain dosage of nano-montmorillonite (3 wt % by virgin asphalt) was adopted to prepare NPMA binders.

**Figure 1:**
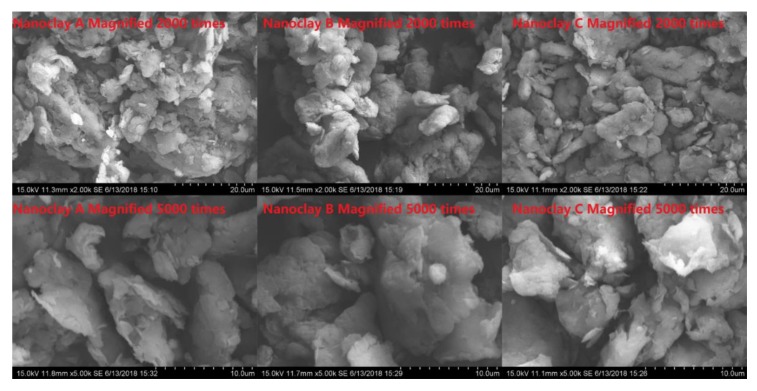
Nano-montmorillonite morphology.

Three different types of nano-montmorillonites, labelled as A, B and C, were applied for asphalt modification. The nano-montmorillonite samples used in this study are organomodified nanoclay particles (provided by the Zhejiang Fenghong Clay Chemical Co., Ltd., Huzhou, China). The nanoclay samples are high-quality montmorillonite with high purity (at least 95% montmorillonite content). Besides, the nano-montmorillonite layer was forgeable due to the large specific surface area (750 m^2^/g) and the unique layered one-dimensional nanostructure and morphology. Among the three nanoclays, A is pure montmorillonite with Na^+^ inorganic group, while B and C are montmorillonites having inorganic groups exchanged with different alkyl ammonium ions. The ranking of their surface hydrophilic properties from high to low is A, B and C. Different from other two-dimensional and three-dimensional inorganic nanoparticles, the specific structure and morphology might lead to excellent mechanical properties, thermal properties, functional properties and physical properties of nano-montmorillonite-polymer modified asphalts. The morphologies of nano-montmorillonites were presented in Figure 1, and the physical properties of different nano-montmorillonites were shown in Table 1. Table 2 detailed the information of each test sample.

### 2.2. Testing Program

The conventional physical properties, including penetration and softening point, were selected as the indicators for the general properties of test binders. The workability was evaluated using a Brookfield viscometer (RVD VII+) through measuring rotational viscosities of asphalt specimen at three different temperatures. 

The rheological properties of modified binders were characterized using a dynamic shear rheometer (DSR, Malvern Kinexus Lab+, Malvern analytical Company, UK). The Superpave rutting factor (G*/sin *δ*) test and multiple stress creep recovery (MSCR) tests were conducted to evaluate the high temperature rutting resistance of asphalt samples, while the intermediate temperature fatigue resistance was analyzed through the Superpave fatigue factor (G*sin *δ*) test and linear amplitude sweep (LAS) test. The test binders for the MSCR test were aged by the standard rolling thin film oven (RTFO) process, while those for the fatigue test were aged by both RTFO and pressure aging vessel (PAV) processes. The bending beam rheometer (BBR) test was also performed to evaluate the low temperature cracking resistance performance of the RTFO + PAV aged samples. 

The storage stability of modified binders was quantitatively analyzed through characterizing the differences in softening point [16,17], complex moduli [24,25,26] and absorbance ratio of CRM/SBR/SBS in the infrared spectrum between the top and bottom portions of the sample after storing. For FTIR tests, the test binder with a thickness of approximately 1 mm was placed in a transmission holder and scanned in order to obtain infrared spectroscopy ranging from 4,000 to 400 cm^−1^. According to ASTM 7173 [27], to simulate the high temperature storing process in the laboratory, about 70 g of hot asphalt was poured into an aluminum tube with a diameter of 25 mm. Before cutting the tube into three equal parts horizontally, it was being stored at 163 °C for 48 h followed by cooling down at −5 °C.

To investigate the modification mechanism of nano-montmorillonite on storage stability, X-ray diffraction (XRD) tests were conducted to investigate the layer gap distance variation. Table 3 shows the detailed information of conducted tests in this study.

## 3. Results and Discussion

### 3.1. Physical Properties

**Figure 2:**
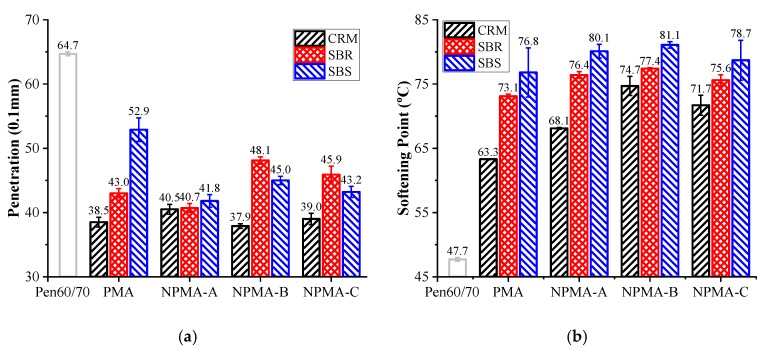
Physical properties of asphalt binders: (**a**) Penetration; (**b**) Softening point.

Figure 2 presents the penetration and softening point results of test binders. It is noted that the polymer-modified asphalts had a higher softening point and a lower penetration, which indicates that the incorporation of CRM/SBR/SBS led to superior performance at high temperature and higher stiffness respectively. It is also observed that the incorporation of nano-montmorillonite further decreased the penetration of SBS-0, while had insignificant effect on CRM-0 and SBR-0. It indicates that the stiffness of SBS modified asphalt binder is more sensitive to nano-montmorillonite compared to the other polymer modified asphalt binders. Different from the penetration results, adding nano-montmorillonite increased the softening points of all modified asphalts.

### 3.2. Workability

**Figure 3:**
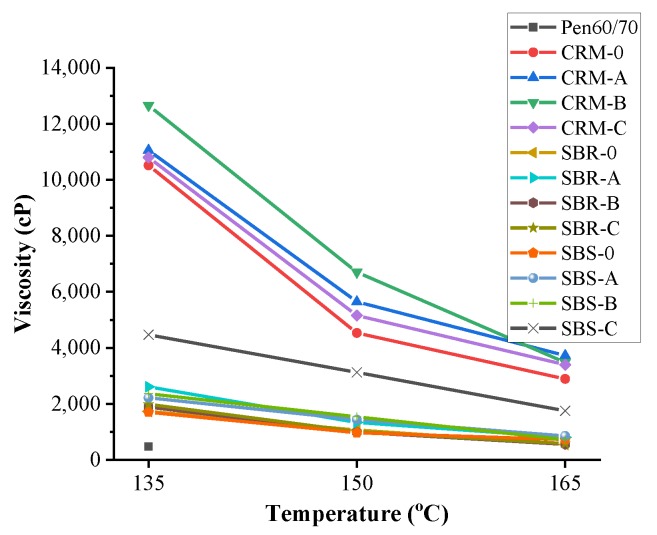
Rotational viscosity test results.

Figure 3 presents the workability results, which were evaluated by the Brookfield rotational viscosity tests. The higher the viscosity value was, the worse workability the test binder had. According to previous studies [28,29,30], poor workability is one of the most critical concerns limiting the spread of asphalt rubber. As expected, CRM modified asphalt binders had extremely higher viscosities than other test binders at all temperatures. Besides, the viscosities of SBR/SBS modified binders except SBS-C were below 3000 cP at 135 °C, which indicates the mixtures with these binders can be compacted according to the AASHTO (American Association of State Highway and Transportation Officials) specification.

### 3.3. Rutting Performance

**Figure 4:**
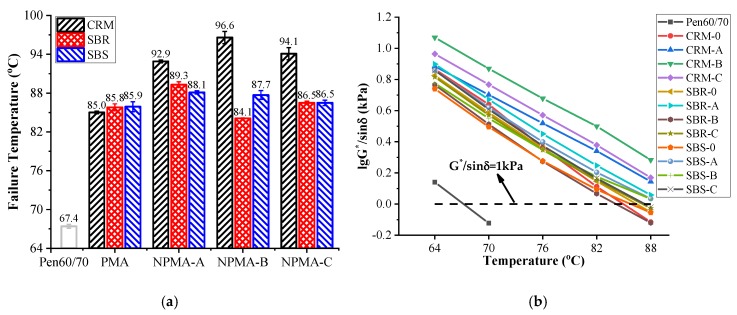
Superpave rutting factor test results: (**a**) Failure temperatures; (**b**) Logarithm of G*/sin δ values.

Figure 4b shows the rutting factor (G*/sin *δ*) values at different temperatures. The starting temperature was set as 64 °C, then the test temperature was automatically increased by 6 °C until the rutting factor was below 1.0 kPa (the critical value for unaged binders). Figure 4a presents the critical temperature results. The higher the critical temperature was, the superior rutting resistance the test binder had. As expected, all of the three polymer modifiers led to much higher critical temperatures than virgin asphalt. It is noted that adding nano-montmorillonite had insignificant effect on SBR-0 and SBS-0. However, the incorporation of nano-montmorillonite further enhanced the rutting resistance of CRM-0, indicating that nano-montmorillonite worked much better with CRM modified binders than SBR/SBS modified binders in terms of rutting resistance.

The MSCR test results were presented in Table 4. Lower *J*_nr_ values and higher % Recovery values refers to superior pavement rutting resistance performance. It is noted that SBS modified binders with nano-montmorillonite and all CRM modified binders did not meet the requirement of AASHTO specification, i.e., <75%. This can be attributed to the extremely low *J*_nr_ value at 0.1 kPa. Consistent with the G*/sin *δ* results, the CRM modified binders exhibited the best rutting property for its lower *J*_nr_3.2 value compared to asphalt binders modified with SBS or SBR. 

### 3.4. Fatigue Performance

**Figure 5:**
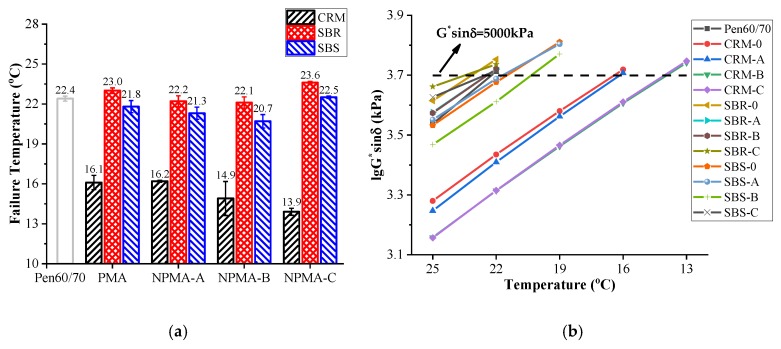
Superpave fatigue factor test results: (**a**) Failure temperatures; (**b**) Logarithm of G*sin *δ* values.

The fatigue critical temperature results are presented in Figure 5a, while the variation of fatigue factor (G*sin δ) with temperatures are shown in Figure 5b. Similar to the G*/sin *δ* test process, the starting temperature was set as 25 °C, then the test temperature was automatically decreased by 3 °C until the fatigue factor exceeded 5,000 kPa. According to the AASHTO specification, lower critical temperature indicates better fatigue resistance performance. It is noted that incorporating CRM effectively decreased the failure temperatures of neat asphalt by 6.3 °C. What’s more, the critical temperatures of CRM-A and CRM-B were 1.2 and 2.2 lower than that of CRM-0, which indicates that the application of nanoclays A and B can further enhance the fatigue performance of CRM-0. However, the incorporation of SBR and SBS had insignificant and even limited negative effects on the fatigue performance of base binder.

**Figure 6:**
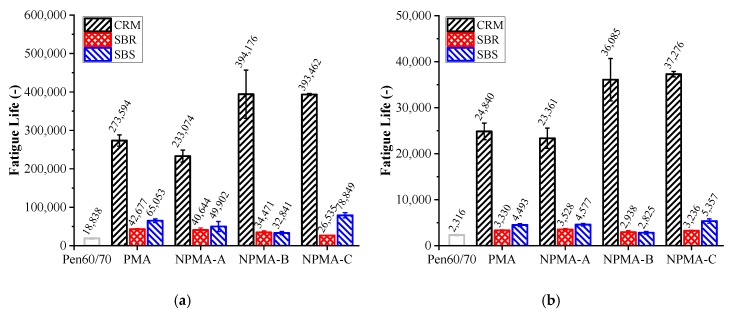
LAS test results: (**a**) Applied strain of 2.5%; (**b**) Applied strain of 5.0%.

Figure 6a,b present the fatigue lives (*N_f_*) of LAS tests at 2.5% and 5.0% applied strain levels, respectively. The higher the fatigue life was, the better fatigue cracking resistance the test specimen had. As shown in Figure 6a, it is noted that the *N_f_* of CRM-0, SBR-0 and SBS-0 was 14.5, 2.3 and 3.5 times that of neat asphalt, respectively. This indicates a different test result from the G*sin δ test results that all modified binders performed better than neat asphalt. According to previous studies [31,32], LAS was proven to be a more reliable method for characterizing fatigue performance of asphalt binders. Therefore, it is believed that CRM exhibited outstanding performance in enhancing fatigue resistance performance, while SBR and SBS had limited positive effect on the fatigue performance of neat asphalt. However, the conclusion of fatigue performance is recommended to be validated by mixture tests like the indirect tensile fatigue (ITFT) test and four-point bending beam (4PB) test.

### 3.5. Low Temperature Cracking Performance

Table 5 presents the stiffness and m-values of test binders determined by the BBR test. To meet a specific requirement of AASHTO T313, the stiffness value should be less than 300 MPa, and the m-value should be higher than 0.3. A low-temperature cracking was more likely caused by higher stiffness values. As expected, the incorporation of CRM/SBR/SBS succeeded in enhancing the cracking resistance of neat asphalt by decreasing the stiffness value. Among three PMA binders, the best low temperature cracking resistance was obtained by CRM-0 for the lowest stiffness. It can also be seen that the incorporation of nano-montmorillonites further decreased the stiffness values of all PMA binders. Eventually, all modified binders were compliant with the requirement at −12 °C. Therefore, all three kinds of modifiers and three nano-montmorillonites can contribute to the application of asphalt pavement in colder regions. It is worth mentioning that recent studies show that the cooling period, temperature and medium may exhibit certain influence on the results of low temperature performance [33]. More accurate and reliable methods for the low temperature performance evaluation of PMA will be investigated in future studies [34].

### 3.6. Storage Stability

#### 3.6.1. Softening Point Difference

**Figure 7:**
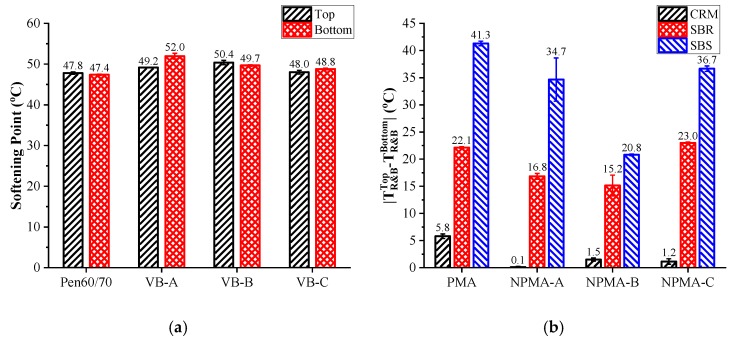

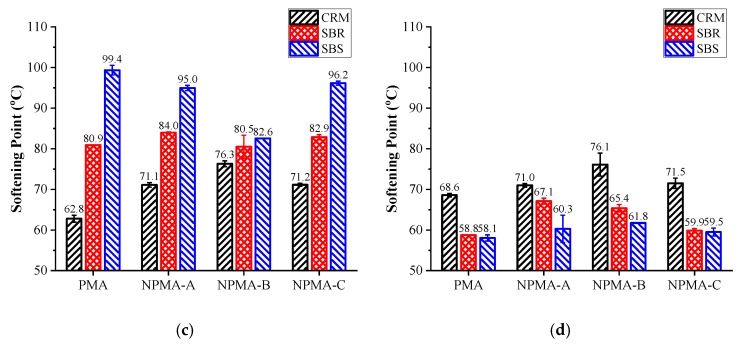
Softening point difference results: (**a**) Nano-montmorillonite modified asphalt; (**b**) D-values of NPMAs; (**c**) Softening points of top sections; (**d**) Softening points of bottom sections.

Figure 7a shows the storage stability results of nano-montmorillonite-modified asphalt based on softening point difference. Smaller difference value (D-value) of softening points indicates superior storage stability. According to Stoke’s law, the nano-montmorillonites tended to sink for its higher gravity (1.7–1.8) compared to virgin asphalt, regardless of its nanostructure, while the D-values of all NMAs met the requirement of AASHTO D5892, i.e., <2.5 °C. Additionally, the softening point difference values of VB-B and VB-C, which were smaller than 0.7 °C, exhibited an extremely stable dispersion of nano-montmorillonite in liquid asphalt. One possible reason may be that the colloidal size and the intercalated layer structure of nano-montmorillonite stopped the process of sinking. The colloidal size with adequate surface area can make the solid particles (nano-montmorillonite) move randomly in asphalt fractions rather than directly ascend (or descend) in vertical direction [35]. What’s more, the stable disperse of nano-montmorillonites in virgin asphalt can also be attributed to the penetration of the asphalt fractions into the nano-montmorillonite layers, which modified the original structure to intercalated or exfoliated structure within asphalt components [17,21].

Figure 7b shows the D-values of modified binders, and Figure 7c,d present the softening point results of top and bottom sections after high-temperature storing, respectively. It is noted that the softening point difference value of SBR-0 was 16.3 °C higher than CRM-0, but 19.2 °C lower than SBS-0. According to the softening point results, this may be caused by the different modification effect of modifiers on the softening point, thus it is believed that all selected PMA binders had poor storage stability. For CRM and SBS modified binders, all three nano-montmorillonites decreased such differences. However, only Nanoclays A and B enhanced the storage stability of SBS-0. Comprehensively considering all test results, Nanoclay B seemed to work best on enhancing the storage stability of polymer-modified asphalt for the lowest D-values for SBR and SBS modified asphalt and the acceptable result for CRM-modified asphalt.

#### 3.6.2. Complex Shear Modulus

According to previous studies [36,37], the complex shear modulus seemed to be a more reliable parameter to investigate the component variation of test binders. Therefore, the separation index (SI, Equation (2)) was proposed based on the complex modulus results by strategic highway research program (SHRP).

(2) $$SI=\left(\frac{Max(G_{Top}^{*},G_{Bottom}^{*})-G_{Avg}^{*}}{G_{Avg}^{*}}\right)\times 100,$$

where $G_{Top}^{*}$ and $G_{Bottom}^{*}$ are the complex shear modulus at 25 °C at a frequency of 10  rad/s of the bottom and top parts after storage, and $G_{Avg}^{*}$ is the average of $G_{Top}^{*}$ and $G_{Bottom}^{*}$.

Table 6 presents the SI values at 25 °C (a typical intermediate temperature), 64 °C (a typical high temperature) and 82 °C (the rutting failure temperature for all PMA binders). It is noted that the SI value of SBR-0 was higher than CRM-0 but lower than SBS-0 at both intermediate and rutting failure temperatures, which is consistent with the results of softening point difference. After mixing with nano-montmorillonite, the *SI* value of CRM-0 at 25 °C and SBR-0 at 82 °C was significantly decreased, while there were no similar findings with SBS-modified binders. Therefore, a more efficient parameter was recommended. A best linear fit was firstly applied to obtain the temperature sensitivity parameter *k* (the slope of Equation (3)). Equation (2) was then used to calculate the separation index of *k*, i.e., *SI_k_*. As shown in Table 7, it is noted that the storage stability of CRM-0 can be effectively enhanced by all selected nano-montmorillonites. Besides, the Nanoclay B worked well on all PMA binders, which was consistent with the softening point difference tests.

(3) $$\log\;G^{*}=kT\;+\;b$$

#### 3.6.3. Fourier Transform Infrared Spectroscopy

**Figure 8:**
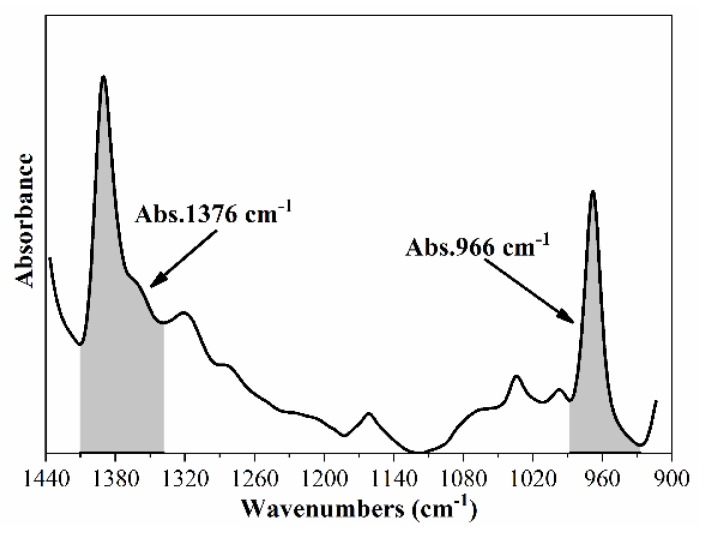
Calculation of area under specific peaks.

According to previous studies [38,39], the Fourier transform infrared (FTIR) tests can be used to evaluate the polymer content in modified asphalt. Infrared spectroscopy ranging from 4000 to 400 cm^−1^ was obtained by scanning using an FTIR spectrometer. In this study, the specific peak at 966 cm^−1^ (caused by out-of-plane bending *γ*CH_3_ vibration of trans-butadiene) was selected as a typical peak of CRM/SBR/SBS, while the peak at 1376 cm^−1^ (caused by in-plane bending vibration of *δ*CH_3_) was selected for virgin asphalt. Figure 8 shows the area of specific peak (Abs. 966 cm^−1^ and Abs.1376 cm^−1^) under the FTIR curve. Then, the absorbance ratio (RA = Abs. 966 cm^−1^/ Abs.1376 cm^−1^) was calculated to present the CRM/SBR/SBS content. A larger absorbance ratio indicates a higher polymer content in the test sample.

Table 8 present the FTIR results of the original binders and their corresponding top and bottom sections after storing. It is noted that the difference in RA values between top and bottom sections of CRM-0 was the largest among three PMA binders, which may indicate that the CRM-0 had the worst storage stability. Different from the other two tests, all types of nano-montmorillonites led to enhanced storage stability of the PMA binders. It can also be seen that nano-montmorillonites worked best on rubber-modified asphalt among three polymer modifiers, which was consistent with the softening point difference and complex shear modulus tests. What’s more, Nanoclay B exhibited the best modification on storage stability among three nano-montmorillonites.

### 3.7. Mechanism Investigation

The XRD tests were used to investigate the modification mechanism of nano-montmorillonites by determining their corresponding variation of layer distance when incorporated into polymer-modified asphalt. Based on the XRD analysis, the basal interlayer spacing (*d*) can be calculated from the first strong peak in the XRD spectra by means of the following equation:

(4) 2dsinθ=λ

**Figure 9:**
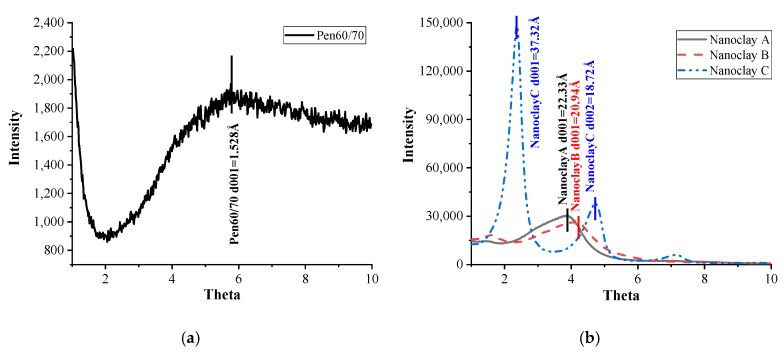

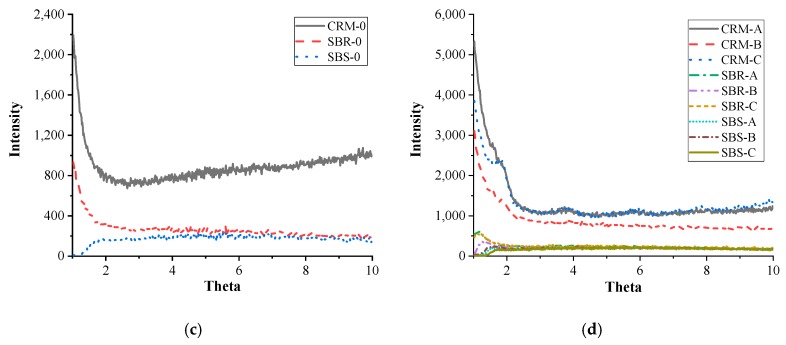
XRD test results: (**a**) Virgin asphalt; (**b**) Nano-montmorillonites; (**c**) Polymer-modified asphalt; (**d**) Nano-montmorillonite-polymer modified asphalt.

Figure 9a shows *d* value of virgin asphalt, while the XRD results of nano-montmorillonites were presented in Figure 9b. It is noted that the d001 of virgin asphalt was quite small in the selected angle ranging from 1° to 10°. Among three nano-montmorillonites, Nanoclays A and B have only one peak while Nanoclay C has two peaks at different positions. The gap distance of Nanoclay C was the largest, while that of Nanoclay B was smallest. In mixed asphalt, the role of the nano-montmorillonite can be characterized according to the distance between the clay plates [23]. Specially, if the distance between the clay plates remain the same, the nano-montmorillonite act like regular particular fillers. In that case, the polymer cannot enter the layer structure of nano-montmorillonites. Conversely, an increased distance indicates the establishment of intercalated structures due to the penetration of polymer chains into the nano-montmorillonites. 

Figure 9c,d present the XRD results of PMA binders and NPMA results, respectively. It is noted that the clay interlayer diffraction peak can be observed at about 1°–2° in the results of NPMA binders, which shifted towards lower angles compared to the results of their corresponding nano-montmorillonites [24]. Nevertheless, there were no peaks noticed in the XRD spectra of PMA binders. Table 9 summarizes the *d* value results of nano-montmorillonite before and after being incorporated into modified asphalt. It is noted that the layer distance of nano-montmorillonite in the SBR-modified asphalt was the largest, while that in CRM-modified asphalt was the smallest regardless of the nano-montmorillonite type.

**Figure 10:**
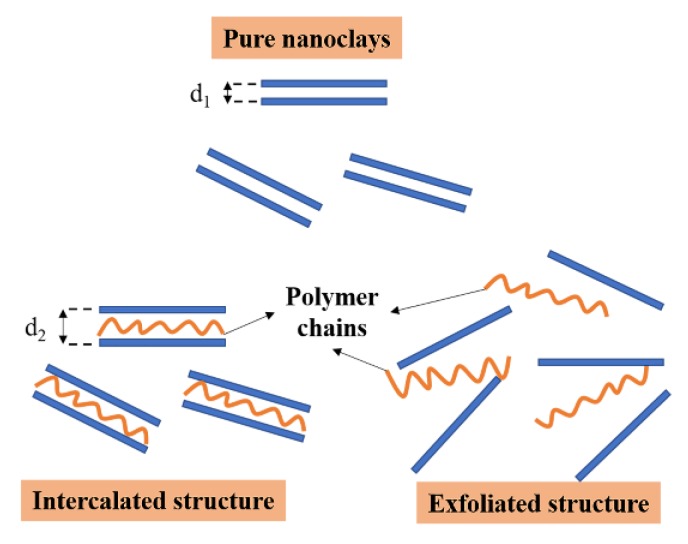
Schematic illustration of intercalated and exfoliated structure.

By incorporating the colloid theory and the experimental results, the enhancement mechanism of nano-montmorillonites on storage stability can be explained as follows. Virgin asphalt is considered a dynamic colloidal system within which high molecular weight asphaltene micelles suspended in the lower molecular weight oily medium (maltenes) [40]. Since the layer distance of nano-montmorillonite did increase in the interaction process with polymers, the polymer chains penetrated the nanolayers were believed to belong to maltenes (in CRM-modified binders), SBR and SBS, respectively. The nano-montmorillonite structure became an intercalated structure rather than an exfoliated structure (Figure 10). With nano sizes and layer structures, the nano-montmorillonites do not settle down easily in hot asphalt even though their densities are larger than that of virgin asphalt. Additionally, with smaller layer distance change, the bonds between polymers and nano-montmorillonite were considered stronger due to Van der Waals forces between nano-montmorillonite layers. Eventually, the existence of nano-layers slowed down the precipitation of polymer modifiers, which results in a more stable disperse of the modifier in virgin asphalt, therefore improving the storage stability of PMA binders.

## 4. Conclusions

This study evaluated the effect of nano-montmorillonite on the properties of modified asphalt with different polymers including CRM, SBR and SBS. Rheological and chemical tests were conducted to characterize the rheological properties of obtained NPMA binders and reveal the modification mechanism of nano-montmorillonite on the storing performance. According to test results, the findings are obtained as follows.

- Adding nano-montmorillonite slightly improved the viscosity of PMA binders, and had insignificant effects on their rheological properties.
- The incorporation of all three types of nano-montmorillonites effectively alleviates the storage stability concern of CRM-0, while having a limited positive effect on SBR-0 and SBS-0.
- Nanoclay B, pure montmorillonite with Hydroxyl organic ammonium, exhibited the most obvious effect in improving the storage stability of the three selected PMA binders.

This study suggests that nano-montmorillonite is a promising modifier to alleviate the storage stability concern for asphalt with polymer modifiers. Future studies will focus on investigating the interaction mechanism among nano-montmorillonite, virgin asphalt and different modifiers, as well as the effects of nano-montmorillonites on the engineering performance of asphalt mixture.

## Figures and Tables

**Table 1:** Properties of nano-montmorillonite additives.

ID	Nanoclay A	Nanoclay B	Nanoclay C
Modified method	Pure MT with Na^+^ inorganic group	Hydroxyl organic ammonium	Double alkyl ammonium
Montmorillonite content	96~98%		
Specific gravity	1.8	1.8	1.7
Bulk gravity	<0.3	≤0.3	≤0.3
Hydrophilic	Medium	Strong	Poor
X-ray d001	2.24 nm	2.09 nm	3.73 nm
Applicable polymer	PE, PP, PVC	N/A	PP and other thermos plasticity polymers

**Table 2:** Composition of different modified asphalt samples.

Binder Type	Sample ID	Type of Modifier	Dosage of Modifier	Type of Nano-Montmorillonite
Virgin asphalt	Pen60/70	N/A	N/A	N/A
NMA	VB-A	N/A	N/A	A
	VB-B	N/A	N/A	B
	VB-C	N/A	N/A	C
PMA	CRM-0	Crumb rubber	20 wt %	N/A
	SBR-0	Styrene-butadiene-rubber	7 wt %	N/A
	SBS-0	Styrene–butadiene-styrene	6 wt %	N/A
NPMA-A	CRM-A	Crumb rubber	20 wt %	A
	SBR-A	Styrene-butadiene-rubber	7 wt %	A
	SBS-A	Styrene–butadiene-styrene	6 wt %	A
NPMA-B	CRM-B	Crumb rubber	20 wt %	B
	SBR-B	Styrene-butadiene-rubber	7 wt %	B
	SBS-B	Styrene–butadiene-styrene	6 wt %	B
NPMA-C	CRM-C	Crumb rubber	20 wt %	C
	SBR-C	Styrene-butadiene-rubber	7 wt %	C
	SBS-C	Styrene–butadiene-styrene	6 wt %	C

**Table 3:** Details of the laboratory test.

Performance	Tests	Aging Level	Specification/Standard	Temperature
General properties	Penetration	unaged	ASTM D5	25 °C
	Softening point		ASTM D36	N/A
Workability	Rotational viscosity	unaged	AASHTO T316	135 °C, 150 °C and 165 °C
Rutting resistance	Rutting factor (G*/sin *δ*)	unaged	AASHTO M320	64–88 °C
	MSCR	RTFO aged	AASHTO MP19-10	64 °C
Fatigue resistance	Fatigue factor (G*sin *δ*)	RTFO + PAV aged	AASHTO M320	25–13 °C
	LAS		AASHTO TP101	25 °C
Storage stability	Softening point	unaged	ASTM D36	N/A
	Complex shear modulus		AASHTO M320	25 °C, 64 °C and 82 °C
	FTIR		N/A	25 °C
Low temperature cracking resistance	BBR	RTFO + PAV aged	AASHTO T313	−6 °C, −12 °C and −18 °C
Internal layer distance of nano-montmorillonite	XRD	unaged	N/A	25 °C

**Table 4:** MSCR test results.

Binder Type	*J*_nr_		*J*_nr_% Diff	% Recovery	
	0.1 kPa (kPa^−1^)	3.2 kPa (kPa^−1^)		0.1 kPa (kPa^−1^)	3.2 kPa (kPa^−1)^
Pen60/70	3.988	4.586	15.0	2.1	−0.5
CRM-0	0.019	0.098	402.6	91.6	63.0
CRM-A	0.034	0.168	392.7	89.0	55.8
CRM-B	0.013	0.090	608.0	94.6	67.1
CRM-C	0.023	0.141	518.4	92.0	59.5
SBR-0	0.360	0.417	15.9	34.0	40.5
SBR-A	0.202	0.310	53.7	67.6	54.9
SBR-B	0.310	0.447	44.3	59.6	50.4
SBR-C	0.320	0.463	44.5	52.1	41.8
SBS-0	0.217	0.382	76.2	68.5	51.7
SBS-A	0.060	0.269	350.1	90.5	64.1
SBS-B	0.096	0.300	213.0	88.0	68.1
SBS-C	0.093	0.300	221.9	86.9	63.5

**Table 5:** BBR test results.

Binder Type	−6 °C		−12 °C		−18 °C	
	Stiffness (MPa)	m-value	Stiffness (MPa)	m-value	Stiffness (MPa)	m-value
Pen60/70	N/A	N/A	280	0.280	541	0.199
CRM-0	84	0.362	172	0.355	330	0.205
CRM-A	62	0.388	117	0.332	235	0.277
CRM-B	58	0.406	103	0.347	213	0.268
CRM-C	62	0.395	109	0.339	256	0.247
SBR-0	107	0.339	203	0.308	347	0.192
SBR-A	89	0.356	188	0.322	352	0.186
SBR-B	84	0.350	180	0.310	308	0.220
SBR-C	80	0.352	173	0.324	316	0.215
SBS-0	97	0.360	175	0.317	316	0.208
SBS-A	66	0.423	143	0.342	275	0.255
SBS-B	74	0.426	155	0.359	289	0.238
SBS-C	70	0.440	140	0.336	260	0.265

**Table 6:** Complex shear modulus results.

Binder Type	25 °C			64 °C			82 °C		
	Top (kPa)	Bottom (kPa)	SI (%)	Top (kPa)	Bottom (kPa)	SI (%)	Top (kPa)	Bottom (kPa)	SI (%)
CRM-0	1458.138	718.015	34.0	10.888	10.838	0.2	2.576	2.715	2.6
CRM-A	1065.133	1008.977	2.7	10.690	10.710	0.1	2.905	2.895	0.2
CRM-B	1062.840	961.900	5.0	14.545	15.166	2.1	4.143	4.242	1.2
CRM-C	886.238	970.832	4.6	11.611	12.159	2.3	3.102	3.370	4.1
SBR-0	464.028	3409.039	76.0	4.869	9.142	30.5	1.846	1.076	26.4
SBR-A	395.050	3039.114	77.0	4.456	9.686	37.0	1.731	1.489	7.5
SBR-B	366.635	2631.595	75.5	3.928	7.516	31.4	1.366	1.125	9.7
SBR-C	328.371	5072.979	87.8	5.790	12.139	35.4	2.477	1.516	24.1
SBS-0	160.889	1905.294	84.4	4.393	4.106	3.4	2.557	0.592	62.4
SBS-A	190.650	2231.642	84.3	7.319	5.218	16.8	4.791	0.882	68.9
SBS-B	145.732	2441.973	88.7	1.494	7.015	64.9	0.534	1.089	34.2
SBS-C	185.006	2945.014	88.2	8.393	7.067	8.6	5.774	0.972	71.2

**Table 7:** Temperature sensitivity evaluation results.

Modifiers	CRM			SBR			SBS		
	k_Top_	k_Bottom_	SI_k_	k_Top_	k_Bottom_	SI_k_	k_Top_	k_Bottom_	SI_k_
N/A	−0.0493	−0.0432	6.6	−0.0435	−0.0621	17.6	−0.0329	−0.0626	31.1
Nanoclay A	−0.0460	−0.0456	0.5	−0.0428	−0.0590	16.0	−0.0294	−0.0610	34.9
Nanoclay B	−0.0432	−0.0421	1.2	−0.0439	−0.0601	15.6	−0.0441	−0.0598	15.2
Nanoclay C	−0.0439	−0.0441	0.2	−0.0385	−0.0627	23.9	−0.0277	−0.0621	38.3

**Table 8:** FTIR test results.

Sample ID	Abs. 966 cm^−1^			Abs.1376 cm^−1^			Absorbance Ratio (%)		
	Original Binder	Top	Bottom	Original Binder	Top	Bottom	Original Binder	Top	Bottom
CRM-0	0.554	10.406	1.084	0.021	0.044	0.100	3.746	0.424	9.226
CRM-A	0.731	1.377	0.428	0.047	0.077	0.012	6.384	5.595	2.870
CRM-B	0.815	1.687	1.238	0.042	0.072	0.061	5.199	4.273	4.909
CRM-C	0.541	0.733	1.316	0.032	0.037	0.065	5.869	5.110	4.971
SBR-0	6.415	2.625	2.690	0.496	0.360	0.095	7.739	13.701	3.530
SBR-A	4.597	4.921	3.350	0.307	0.492	0.176	6.675	9.990	5.265
SBR-B	3.729	6.747	5.551	0.326	0.674	0.319	8.745	9.988	5.747
SBR-C	6.189	7.049	2.572	0.596	1.065	0.317	9.625	15.109	12.318
SBS-0	2.392	4.730	9.357	0.381	1.949	0.716	15.938	41.201	7.650
SBS-A	3.377	3.740	6.663	0.566	1.478	0.529	16.773	39.522	7.939
SBS-B	3.907	3.656	8.985	0.518	1.323	1.341	17.231	36.193	14.926
SBS-C	1.506	4.512	8.391	0.300	1.929	1.102	20.327	42.762	13.132

**Table 9:** Layer distance of nano-montmorillonite before and after mixed with asphalt.

Nano-Montmorillonite Type	Original Layer Distance (Å)	Measured Layer Distance in PMA (Increment) (Å)		
		CRM	SBR	SBS
Nanoclay A	22.33	46.49 (24.16)	73.28 (50.95)	53.48 (31.15)
Nanoclay B	20.09	48.42 (28.33)	65.41 (45.32)	53.90 (33.81)
Nanoclay C	37.32	48.33 (11.01)	71.71 (34.39)	50.29 (12.97)
